# Microbes and Mental Illness: Past, Present, and Future

**DOI:** 10.3390/healthcare12010083

**Published:** 2023-12-29

**Authors:** Robert C. Bransfield, Charlotte Mao, Rosalie Greenberg

**Affiliations:** 1 Rutgers-Robert Wood Johnson Medical School, Piscataway, NJ 08854, USA; 2 Hackensack Meridian School of Medicine, Nutey, NJ 07110, USA; 3 Invisible International, Cambridge, MA 02138, USA; charlotte@invisible.international; 4 Medical Arts Psychotherapy Associates P.A., Summit, NJ 07901, USA; rgmd@verizon.net

**Keywords:** aggression, anxiety, autism, bipolar, depression, immune, Lyme borreliosis, schizophrenia, suicide, Tuskegee

## Abstract

A review of the association between microbes and mental illness is performed, including the history, relevant definitions, infectious agents associated with mental illnesses, complex interactive infections, total load theory, pathophysiology, psychoimmunology, psychoneuroimmunology, clinical presentations, early-life infections, clinical assessment, and treatment. Perspectives on the etiology of mental illness have evolved from demonic possession toward multisystem biologically based models that include gene expression, environmental triggers, immune mediators, and infectious diseases. Microbes are associated with a number of mental disorders, including autism, schizophrenia, bipolar disorder, depressive disorders, and anxiety disorders, as well as suicidality and aggressive or violent behaviors. Specific microbes that have been associated or potentially associated with at least one of these conditions include *Aspergillus*, *Babesia*, *Bartonella*, Borna disease virus, *Borrelia burgdorferi* (Lyme disease), *Candida*, *Chlamydia*, coronaviruses (e.g., SARS-CoV-2), *Cryptococcus neoformans*, cytomegalovirus, enteroviruses, Epstein–Barr virus, hepatitis C, herpes simplex virus, human endogenous retroviruses, human immunodeficiency virus, human herpesvirus-6 (HHV-6), human T-cell lymphotropic virus type 1, influenza viruses, measles virus, *Mycoplasma*, *Plasmodium*, rubella virus, Group A *Streptococcus* (PANDAS), *Taenia solium*, *Toxoplasma gondii*, *Treponema pallidum* (syphilis), *Trypanosoma*, and West Nile virus. Recognition of the microbe and mental illness association with the development of greater interdisciplinary research, education, and treatment options may prevent and reduce mental illness morbidity, disability, and mortality.

## 1. Introduction

As human beings, we are interdependent upon both the microbiota within us and those present within our environment. In 2007, the United States National Institutes of Health Human Microbiome Project was established to study the microbial communities that live in and on our bodies, with the goal of elucidating their role in human health and disease [1]. With advancing technology, there is greater recognition that infectious diseases contribute to not only acute but also chronic illness, both physical and mental. Research from the United States Centers for Disease Control and Prevention (CDC) has recognized “that non-communicable chronic diseases can stem from a variety of infectious agents” [2]. Identifying these relationships between pathogens and illnesses can significantly impact human health, both acutely and chronically. Knowledge of these processes creates opportunities for prevention or early invention. The end goal is to reduce or eliminate the impact of illness, especially chronic disease. Scientific evidence demonstrates more support for the role of infectious agents in cancers, immune-mediated syndromes, neurodevelopmental disorders, and other chronic conditions [2,3]. To benefit from this research, clinicians, public health practitioners, and policymakers need to recognize that many chronic diseases may have infectious origins [2].

It is recognized that some infectious diseases can play a significant role in the etiology of neuropsychiatric disturbances. There is little debate that syphilis can cause the development of symptoms of various mental illnesses [4]. Some suspect and others recognize that the novel SARS-CoV-2 virus that is responsible for COVID-19 can contribute to mental illnesses [5]. There exists some uncertainty as to whether some other infections, such as Lyme borreliosis/tick-borne disease, are associated with neuropsychiatric disorders. However, there are over 500 journal articles supporting this association [6,7,8,9,10,11,12,13] (Supplementary S1).

A number of barriers exist that impede medical progress. The obstacles include the following: (1) most psychiatrists have limited knowledge of infectious diseases, (2) most infectious disease specialists have limited awareness of psychiatric diseases, and (3) most physicians have a very limited understanding of psychoneuroimmunology. There are also multiple controversies. How significant are microbes vs. other contributors to the development of mental illness? Etiologically, how important are prior infections vs. current, latent, or active stealth infections? How do infections by multiple pathogens interact with the disease and affect the clinical presentation? How reliable is the present state of laboratory testing in determining the presence or absence of a contributory pathogen? What are the underlying pathophysiological processes involved? Achieving a better understanding of the nature of the association and the possible contribution of infections to the development of mental illness potentially opens up new opportunities for prevention and treatment.

## 2. Materials and Methods

The review identified and evaluated the literature from electronic databases, including PubMed and Google Scholar, for relevant information on the topics previously identified. The references discovered in searches were also reviewed for additional relevant references. In addition, references were also drawn from the libraries previously accumulated by the three authors. The inclusion criteria were peer-reviewed articles taken from all time periods, all articles except editorials, without age or gender subject restriction. A few review articles were included. Most of the references included were listed on PubMed. The included references were fully read by at least one author. A formal PRISMA analysis of the literature was considered but was not performed since there was an overwhelming number of citations related to microbes and mental illness.

The review of the association between microbes and mental illness was performed in multiple separate stages. As a starting point, we examined the literature tracing the evolution of thinking on the etiology of mental illness. Definitions of relevant terms were then clarified. Next, disease models to understand the causes of diseases were defined. A list of infectious agents with potential psychiatric manifestations was developed (Table 1). Then, a review of examples of mental conditions potentially associated with infections was performed. This review included the five mental illnesses with the greatest psychiatric disability (autism spectrum disorders, schizophrenia, bipolar disorders, depressive disorders, and anxiety disorders) [14] and two behaviors of particular concern in psychiatric patients (suicidality and aggressive or violent behavior) (Table 2). Next, the disease models, pathophysiology, and clinical considerations were summarized. This information was then a foundation to review five different infectious diseases associated with mental illness: syphilis; toxoplasmosis; COVID-19; Lyme borreliosis and associated diseases; and group A streptococcal infections and pediatric autoimmune neuropsychiatric disorders associated with streptococcal infections/pediatric acute-onset neuropsychiatric syndrome (PANDAS/PANS). Syphilis has historical significance. The other illnesses have been the subject of more recent interest.

## 3. Results

### 3.1. Overview

What is the evidence for an association between infections and mental illness? Addressing this question requires a multifaceted approach. We shall utilize information from different perspectives to address this question. This includes historical perspectives, definitions of relevant terms, disease models, awareness of infections associated with mental illnesses, examples of mental conditions potentially associated with infections, pathophysiology, and clinical considerations. This information shall then be applied to a more detailed analysis of five quite different infectious diseases associated with mental illnesses.

#### 3.1.1. History of Mental Illness

The earliest evidence of the recognition of mental illness is the discovery of trephination in skulls dating back thousands of years. Trephination is the removal of a small area of the skull using an auger, bore, or saw. This practice was likely to relieve headaches or mental illness based upon the belief in demonic possession [284]. References to what appear to be mood disorders are noted in the writings of ancient Greco-Roman physicians. The oldest written description of a schizophreniform type of illness was written in the *Ebers Papyrus* from the time of the Egyptian Pharaohs. The *Ebers Papyrus* is a compendium of ancient Egyptian Medical Papers listing treatments for multiple illnesses. The description was found in a section called Book of the Heart [285]. Ancient Egyptians believed that the mind and the heart were similar to each other. To the Egyptians, “Physical illnesses were regarded as symptoms of the heart and the uterus and originating from the blood vessels or from purulence, fecal matter, a poison or demons” [285].

In the first century, Aretaeus of Cappadocia was the first to determine that there was an association between the brain and the two disparate mood states. Plato spoke of two types of mania, “One involving a mental strain that arises from a bodily cause of origin, the other divine or inspired”. Both Hippocrates and Aretaeus tried to prove that, in some cases, melancholia and mania were of biologic origin, not just a mental response as a reaction to situations. When it came to hallucinations, the Hippocratic doctors recognized it as a sign of a medical problem, while most common people still thought it was due to the gods [286].

A commonly held belief was that mental illness was the result of demonic possession, witchcraft, or an angry god [287]. Based upon this belief, witch-hunting resulted in more than 100,000 presumed “witches” being burned at the stake. This practice did not decline until the 17th and 18th centuries [288,289]. There is speculation that the witch trials in Salem, Massachusetts, in the 1600s may have been a response to an epidemic of autoimmune encephalitis in which individuals had thrashing fits with bizarre behavior, possibly caused by an autoimmune process [290]. Hypothesized causes of the bizarre behavior include encephalitis lethargica, Huntington’s chorea, a rye fungus causing anti-*N*-methyl-D-aspartate receptor encephalitis, and Lyme disease [289,291,292]. For centuries, based upon the belief in supernatural forces and demonic possession, the mentally ill were treated very poorly, subjected to physical restraints and solitary confinement in asylums.

In the first part of the 1800s, Wilhelm Griesinger, a German psychiatrist and neurologist, was an active proponent of the theory that “All mental illness is disease of the brain” [293]. From this vantage point, mental disorders were placed more clearly in the biological domain of medicine and no longer in the realm of the mystical or supernatural. Griesinger’s strong belief in this postulate led him to become an advocate for better and more humane treatment of those who were mentally ill in asylums. Philippe Pinel and Dorothea Dix further advanced the concept of humane treatment for the mentally ill in the 1800s [294].

In 1887, the German psychiatrist Emile Kraeplin first described what he called “dementia praecox”, which he believed was a disease of the human brain. In 1908, Eugen Bleuler, a Swiss psychiatrist, changed the name to schizophrenia. Furthering this medicalization of psychiatry in the early 1900s was the theoretical belief that a focal infection was the source of mental illness or brain disorders. 

An American psychiatrist, Henry Cotton, who had undergone some psychiatric training in Europe and was a protégé of the renowned psychiatrist Adolf Meyer at John Hopkins, became a strong advocate of this theory [295]. His basic belief was that eliminating the source of infection was the only way to cure an individual’s mental illness. Cotton served as the medical director and superintendent of the New Jersey State Hospital in Trenton, N.J., from 1907 to 1930. He went on to teach that chronic mental illness was the result of ongoing, latent, and somewhat stealth or unrecognized infections. For him, the proper treatment of a mentally ill individual would be the removal of the initiating and offending infectious site. This often led to the removal of the affected organ. In the name of treatment, teeth were pulled, and the uterus or parts of the gastrointestinal tract or other body parts were surgically extracted. Unfortunately, what initially appeared to be a promising concept lacked sufficient evidence and resulted in the maiming and disfigurement of many individuals. In the end, it became clear that this was a horrific treatment intervention. Although the concept that biologic influences, including infections, were at play in mental disorders indicated thinking in the right direction, Dr. Cotton’s interpretation was disfiguring and barbaric. 

Sigmund Freud recognized the value of acquiring insight into and an understanding of psychodynamic development to better explain motivation. Anna Freud, Erik Erikson, and others further expanded on defense mechanisms and developmental theories.

In the 1950s, “the catecholamine hypothesis of affective disorders” emerged. This theory attributed mood disorders to deficits or excesses of certain catecholamines as the physiologic cause of mental illness [296].

In the late 1970s, the biopsychosocial model of psychiatric illness was developed by Drs. George Engel and John Romano. This approach recognized that there was a combination of biological, psychological, and social contributors to mental illness. 

Understanding the inner workings of the mind and the brain has always been challenging. For its weight, nothing is more complex than the human brain. For many years, the living brain was seen as a mysterious black box that could not be understood. The development of brain imaging technology with computed tomography, magnetic resonance imaging, positron emission technology, single-photon emission technology, and other neuroscience advances allowed the imaging of the anatomy and physiology of the living brain. These techniques improved the capacity to better understand the complexities and brain circuits involved in mental functioning.

Advances in gene technology hastened interest in the genetic causes of mental illnesses. Darwinian or evolutionary medicine recognized that most illness is not caused by disease genes but instead by susceptibility genes interacting with environmental contributors. The environmental contributors include competing organisms, such as microbes [297].

With a greater understanding of genetics came the recognition that the interaction between genes and health is even more complex than previously realized. This awareness has resulted in the recognition of the importance of epigenetic factors. The field of epigenetics, as defined by the CDC, refers to “the study of how your behaviors and environment can cause changes that affect the way your genes work. Unlike genetic changes, epigenetic changes are reversible and do not change your DNA sequence, but they can change how your body reads a DNA sequence” [298]. Focusing only on genes, without attention to epigenetic factors, will only give us part of the genetic picture when studying mental illness. Environmental contributors also play an important role [299].

#### 3.1.2. History of Associating Microbes and Mental Illness

Microbiology began with the lens. It was originally used for millennia for other purposes. The machine to manufacture precision lenses was designed by Leonardo Da Vinci [300,301]. Combining two lenses resulted in the microscope that created the technology to observe both microorganisms and human cells. Hooke and Leeuwenhoek observed microorganisms in the late 1600s [302].

Rudolph Virchow used the microscope to directly visualize anatomical changes in cells and groups of cells when a patient became ill. He proposed that when ill, the whole organism does not get sick. Instead, it is only particular cells or groups of cells that change, thus opening up the new field of cellular biology. The use of a microscope now added the ability to utilize direct visual anatomical changes for identifying diseases. No longer did illnesses need to be diagnosed by clinical symptoms alone. Virchow also coined the term “zoonosis”, indicating the connection between human and animal health, which could be considered the foundation of the concept of One Health or One Medicine [303].

The highly significant work of Robert Koch, Friedrich Loeffler, and Louis Pasteur in the late 1800s led to the discovery that microbes (germs) could cause disease [304]. The science of medicine changed dramatically when the germ theory of disease was proven. This awareness opened up new avenues for exploration. These advances dramatically improved the science of medicine in the 1800s.

As previously noted, theories on the origins of mental illness remained a mix of science, spirituality, and myth for centuries. An early attempt utilizing germ theory in the field of mental health is exemplified in the following report:

“In 1896 Scientific American published an editorial entitled ‘Is Insanity Due to a Microbe?’ Two doctors described how they had injected cerebrospinal fluid of mentally ill patients into rabbits, which later got sick. Subsequently, the rabbits showed behavioral issues. The authors concluded that ‘certain forms of insanity’ could be caused by infectious agents, ‘similar to typhoid, diphtheria and others.”

[305]

Although this early work could explain some diseases, the etiology of mental illnesses remained somewhat puzzling. The recognition of a potential connection between infections and mental symptoms may have been partially based on ongoing observations of mental changes, e.g., psychoses, that occurred with some bacterial illnesses. Throughout the 1800s and well into the 1900s, general paralysis of the insane, also known as general paresis and caused by neurosyphilis, was a significant degenerative mental illness known to psychiatry. In 1913, Hideyo Noguchi found traces of *Treponema pallidum* (the bacteria that causes syphilis) in the brains of deceased general paresis patients who had been hospitalized for mental illnesses at the Central Islip State Hospital for the Insane [20]. In 1943, penicillin became the main treatment for syphilis [306].

After penicillin became an effective treatment for syphilis, there was little attention to the association between infections and mental illness for many years. In the early 1990s, Fallon began writing about the association between Lyme disease and mental illness [13]. In 1996, the Stanley Laboratory of the Johns Hopkins University School of Medicine was founded as a result of the efforts of Robert Yolken and E Fuller Torrey to elucidate and promote research and training on the role of infection and immunity in the etiology of schizophrenia and bipolar disorders. Work at the Stanley Center, other collaborating centers (Karolinska Institute, University of Heidelberg, University of Cologne, University of Pittsburgh, Centre for Register-Based Research in Denmark, Sheppard Pratt, Washington University, University of Cambridge, University of Maryland, Harvard School of Public Health, etc.), and other projects funded by it has resulted in extensive research and many peer-reviewed publications. This improved the understanding of the association between microbes and mental illness [307]. The field has continued to progress since then. Presently, there are a vast number of articles in the peer-reviewed literature addressing different facets of the association between microbes and mental illness [308].

### 3.2. Definitions of Relevant Terms

#### 3.2.1. Microbes and Related Terms

A microbe has traditionally been defined as a very small, living thing that can be seen only with the use of a microscope. There are a number of related terms. The human microbiota is the full array of microorganisms that live on and in humans. The human microbiome is the combined genetic material of the microorganisms that live in and on humans. The pathobiome is the set of human-host-associated organisms (crucially encompassing prokaryotes, eukaryotes, and viruses) associated with reduced (or potentially reduced) health status as a result of interactions between members of that set and the human host.

#### 3.2.2. Health

A generally accepted definition of health is the ability to adapt and to self-manage in the face of social, physical, and emotional challenges [309].

#### 3.2.3. Illness

Illness refers to an unhealthy condition of the body or mind: “The human experience of sickness” [310] and “The innately human experience of symptoms and suffering” [311].

#### 3.2.4. Mental Health

According to the American Psychiatric Association, mental health is a state of mind characterized by emotional well-being, good behavioral adjustment, relative freedom from anxiety and disabling symptoms, and a capacity to establish constructive relationships and cope with the ordinary demands and stresses of life [312]. The World Health Organization defines mental health as “a state of well-being in which the individual realizes his or her own abilities, can cope with the normal stresses of life, can work productively and fruitfully, and is able to make a contribution to his or her community” [313]. In summary, mental health occurs when mental functioning (cognition, emotions, vegetative functioning) reflects the individual’s life situation and facilitates adaptation with the capacity to experience well-being, pleasure, fulfilling relationships, and productive activities; the ability to recognize and contend with adversity; and the mental flexibility to adapt to change.

#### 3.2.5. Mental Illness

The American Psychiatric Association defines a mental disorder as a syndrome characterized by a clinically significant disturbance in an individual’s cognition, emotion regulation, or behavior that reflects a dysfunction in the psychological, biological, or developmental processes underlying mental functioning [312]. In a state of mental illness, mental functioning does not reflect the life situation, and there is an impairment of adaptive capabilities; an impaired capacity to experience well-being, pleasure, fulfilling relationships, and/or productive activities; a diminished ability to recognize and contend with adversity; and/or restricted mental flexibility to adapt to change.

#### 3.2.6. Acute vs. Chronic Illness

Acute illnesses generally develop suddenly and last a short time, often only a few days or weeks. Chronic conditions, by contrast, are long-developing and may worsen over an extended period of time—months to years. Chronic diseases more often can be controlled but not cured. Some define chronic diseases as lasting at least 3 or 6 months. Chronic diseases are defined broadly by the CDC as “conditions that last 1 year or more and require ongoing medical attention or limit activities of daily living or both” [314]. Post-acute is a term that has more recently been used.

### 3.3. Models for Understanding Disease

Illness is caused by an underlying disease process. To better understand the underlying disease process, a variety of approaches can be used.

#### 3.3.1. A Multisystem Approach to Understanding the Cause of Disease

A multisystem approach is useful when analyzing complex issues, such as the cause of disease. It allows for a complex model with many contributors. All systems are interconnected and affect other systems to varying degrees. Furthermore, they are constantly changing and in dynamic balance with each other. Time is a significant dimension, and different effects occur over time. Systems have evolved over the dimension of time. The combination of the systems and evolutionary approaches allows us to organize current information in a much more efficient manner [315].

#### 3.3.2. An Evolutionary or Darwinian Approach to Understanding the Cause of Disease

An evolutionary or Darwinian approach applies the principles of evolutionary biology to help explain problems in medicine and public health and prevent human disease. It explains how disease occurs from the perspective of evolution. From this perspective, disease can be the result of genetic vulnerabilities from the unique path of evolution, design compromises, and competing organisms. Competing organisms can include microbes. “Core principles drawn from evolutionary biology include selection, drift, plasticity, mismatch, cultural practices, trade-offs, life history traits, antagonistic pleiotropy, heterozygote advantage, constraints, biologic defenses, co-evolution (i.e., microbiome), adaptation/maladaptation, novel environments, and the genome-phenome relationship” [299,316].

#### 3.3.3. An Organismal Approach to Understanding Disease

**Figure 1:**
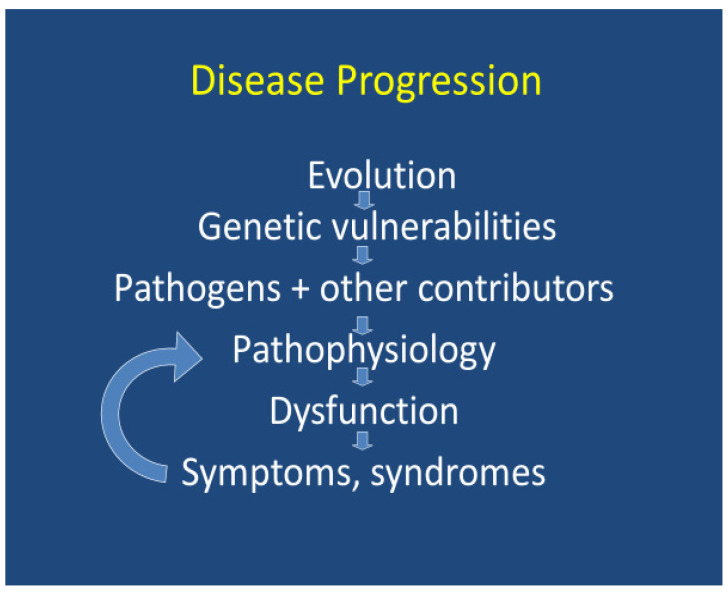
Disease progression. Disease progression evolves over time. The unique path of evolution can create genetic vulnerabilities. Genetic and other vulnerabilities may then interact with pathogens and other disease contributors to begin a pathological process with regulatory dysfunction. The resulting pathological cascade may then lead to symptoms and syndromes (groups of symptoms). The symptoms, and syndromes (e.g., chronic stress, sleep deprivation, immune dysfunction) may further exacerbate disease progression.

Disease results from an interaction of host vulnerabilities and environmental contributors. Host vulnerabilities may be genetic or otherwise, and the environmental contributors may be of infectious or non-infectious origin. While acknowledging that there are many non-infectious contributors associated with mental illness, this article specifically focuses upon the infectious contributors. The infectious and/or non-infectious contributors may then provoke immune activity that can result in the impairment of adaptive mechanisms, resulting in disease progression [139,317,318,319,320,321,322,323,324,325] (Figure 1).

#### 3.3.4. Infections Associated with Mental Illness

A myriad of journal articles address different aspects of the possible connection between infections and mental illnesses. Some mental illness symptoms, such as those associated with delirium, are generally, although not exclusively, associated with the acute phase of infection [326,327]. Viruses and vector-borne diseases have drawn some of the greatest attention. There are at least 320,000 different species of viruses in mammals [328]. Vector-borne diseases are mostly zoonotic, i.e., diseases that are transmitted between species, from animals to humans, or from humans to animals. It appears that vector-borne diseases are increasing, and possible explanations include heightened awareness, climate change, changing ecosystems, globalization, human population growth, and toxic warfare environments [329,330].

A partial list of infectious agents that are associated with potential psychiatric manifestations is found in Table 1. It includes spirochetes, other bacteria, viruses, parasites, and fungi [105].

#### 3.3.5. What Mental Illnesses Are Associated with Specific Microbes?

A systematic review and meta-analysis was previously conducted on mental illnesses associated with SARS-CoV-2 infection [331], and a prior PRISMA analysis was performed on mental illnesses associated with Lyme borreliosis [7].

A search was conducted for specific microbes associated with five mental illnesses with the greatest psychiatric disability and two behaviors of particular concern in psychiatric patients (Table 2).

The microbes identified with these conditions collectively included *Aspergillus*, *Babesia*, *Bartonella*, relapsing fever group *Borrelia*, *Borrelia burgdorferi*, Borna disease virus, *Candida*, *Chlamydia*, SARS-CoV-2 (COVID-19) and other coronaviruses, cytomegalovirus, enterovirus, Epstein–Barr virus, hepatitis C virus, herpes simplex virus, human endogenous retroviruses, human herpesvirus-6, human immunodeficiency virus (HIV), human T-cell lymphotropic virus type 1 (HTLV-1), influenza viruses, measles virus, *Mycoplasma*, *Plasmodium*, rubella virus (congenital), *Shigella*, group A *Streptococcus*, *Taenia solium*, *Toxoplasma gondii*, *Treponema pallidum* (syphilis), varicella zoster virus, and changes in intestinal microbiota composition.

#### 3.3.6. Disease Models

- Acute vs. chronic infections

As noted earlier, the CDC defines a chronic illness as lasting one year or more [314]. The nomenclature regarding acute vs. chronic infections and acute vs. chronic manifestations of infectious diseases can be confusing. Some acute infections can be “hit and run” and cause residual injury that leads to dysfunction and chronic disease, with the illness continuing and sometimes progressing long after the infection is clearly eradicated. Developing mitral valve stenosis following a streptococcal infection is an example of this. On the other hand, infections can also be recognized as being chronic with ongoing symptoms and often disease progression as the pathogen persists. An example of this is seen with syphilis. Not all infections are easily placed into these two categories. Some infections may be acute, then be latent, and then be reactivated at a later time. An example of this is varicella zoster (chicken pox), which can be reactivated as shingles decades later when the patient is in an immunocompromised state. Some infections may suppress and/or evade the immune system, which results in difficulty detecting them by commonly used immune-based testing. These immune-evasive, persistent infections can cause symptoms that are chronically progressive or chronically relapsing and remitting. An example is *Borrelia burgdorferi* [332,333,334,335,336,337]. Persistent or progressive symptoms after commonly used treatment for *Borrelia burgdorferi* infection are viewed by some researchers as caused by a self-perpetuating immune process in the absence of ongoing active infection [338].

- Complex interactive infections

Koch’s initial view of infectious disease is that many human diseases are caused by microbes that exert their effects independently of other microbes, environmental factors, or genes. However, most common human diseases are caused by the interaction of environmental insults and susceptibility genes [339]. Many of the susceptibility genes are diverse determinants of human responses to environmental factors, e.g., infection.

Informative laboratory methods for complex disorders must address both genetic and environmental factors [340].

Some infection-associated symptom presentations are not the result of single pathogens but instead the result of complex interactive infections with multiple infectious agents. These infections could be compared to a foxhunt, in which three different species (humans, horses, and dogs) participate. In this case, the foxhunt would have a very different effect than if only one species were involved. Human immunodeficiency virus infection and acquired immune deficiency syndrome (HIV/AIDS) is one example in which a pathogen (virus) causes immunodeficiency, allowing other microbes to become more pathogenic to the host. This pattern can also be seen with tick-borne diseases, as multiple tick-borne pathogens such as *Borrelia*, *Babesia*, *Anaplasma*, *Ehrlichia*, other *Rickettsia*, *Nematodes*, etc., may be present at the same time [341]. Additional unidentified, non-testable pathogens may also be contributory. The *Borrelia* bacterium has the capacity to cause immunosuppression and immune evasion [332,333,334,335,336,337]. This can result in previously asymptomatic latent infections becoming symptomatic. These previously acquired latent infections may not have been transmitted via tick bites and may include viruses and *Bartonella* and *Mycoplasma* species [342,343,344]. In addition, tick-borne polymicrobial coinfections can have an interactive effect upon the *Borrelia* infection [345,346,347]. SARS-CoV-2 viral infections have also been associated with complex interactive infections. There is anecdotal evidence that COVID-19 infections have resulted in the re-activation of latent *Borrelia* infections [348]. Increased levels of *Borrelia*-specific IgG antibodies strongly correlated with SARS-CoV-2 viral severity and the risk of hospitalization [349]. The human microbiota has been implicated in the development of a variety of mental illnesses, including Alzheimer’s disease, attention-deficit/hyperactivity disorder, anorexia nervosa, autism spectrum disorder, bipolar disorder, major depressive disorder, schizophrenia, and substance use disorders [350]. The human gut microbiome is recognized to have a significant impact upon both health and disease, as well as human metabolism, nutrition, physiology, and immune functioning. An altered gut microbiome is associated with different disease states [138,351]. The human microbiome is not just located in the gut. It can also be located in other parts of the body [350]. Some of the human microbiota can be located deep in connective tissue, where they may be protected from the immune system and attempts at antibiotic treatment [352,353,354].

When there is a complex interactive infection, it is sometimes possible to say that 1 + 1 does not = 2, but instead, 1 + 1 = 11.

- Total load theory

Microbes may also interact with other non-infectious environmental contributors. The initial foundation of this view is based upon the disease triangle, which is a conceptual model showing the interactions between the environment, the host, and an infectious (or abiotic) agent [355]. This concept is addressed in the total load theory. Here, the focus is on how the presence of multiple stressors can result in an increasing number of developmental delays, cognitive problems, behavioral and emotional issues, and other impairments that have been seen in children in recent years. The approach views groups of symptoms as being the result of reaching a tipping point where development is stressed beyond the capacity for healthy adaptation. At that point, the youth shows signs of overload. This state is then manifested as attentional difficulties, developmental delays, mood issues, autoimmune problems, failure to thrive, repeated ear infections, etc. Some of the elements that can lead to developmental issues include birth trauma, pregnancy complications, nutritional deficiencies, frequent ear infections, etc. [356].

### 3.4. Pathophysiology

#### 3.4.1. Is Trauma from Infection or from the Host’s Immune Reaction to the Infection?

Symptoms associated with infectious disease are a result of both the direct effects of the pathogen upon the host and the indirect effects caused by host immune activation. In an acute infection, the early inflammatory (innate immune) response evolves into a humoral (adaptive immune) response. In a chronic infection, there may instead be a persistence of the inflammatory state without effective adaptive immunity, and sometimes with an autoimmune response. This interaction between pathogen and host contributors results in trauma to the host. Microbial contributors to pathophysiology include toxin release, cell penetration, the effects of bacterial lipoproteins, and the incorporation of pathogen genes into the host genome. Host response contributors can include cytokine release, inflammation, inflammation-causing metabolic changes, the effects of bacterial lipoproteins and other pathogen-associated molecular patterns (PAMPs) such as toll-like receptor signaling, the interaction of heat shock proteins with the immune system, oxidative stress, the action of nitric oxide, other cellular responses, and autoimmune reactions [9,12,357,358,359]. Oxidative stress and changes in nitric oxide are also well recognized as being a part of the stress response and the pathogenesis of depression and other mental illnesses [360,361,362].

In addition, infections outside the central nervous system (CNS) can have immune and toxic effects upon the CNS, with molecular mimicry leading to autoantibody formation and cellular immune responses against host neuronal structures, oxidative stress, glutamate excitotoxicity, changes in homocysteine metabolism, mitochondrial dysfunction, and altered metabolism of tryptophan with decreased production of the neurotransmitter serotonin and increased production of neurotoxic and excitotoxic quinolinic acid and kynurenine metabolites [363,364,365,366,367].

The glymphatic system is important in healthy brain functioning and the prevention of the accumulation of neurotoxic cellular waste products [368,369]. A common neuropsychiatric symptom with a microbial infection is some form of insomnia [370]. During sleep, the glymphatic system expands, and our brain contracts as our immune system attempts to eliminate waste and cleanse itself [371]. If a bacterial toxin released by living organisms or perhaps dead remnants of microbes is not cleared, destructive secretions or ongoing immune stimulation can result in neuroinflammation and neural degeneration. This then has the potential to contribute to cognitive, behavioral, and emotional difficulties.

These pathophysiological processes are hypothesized to result in neuropsychiatric symptoms [12,364,365,366,372,373,374,375]. Bacterial infections are associated with many autoimmune diseases involving chronic inflammation and demyelination [376]. The pathophysiology of how these mechanisms impact the brain is addressed in the field of psychoneuroimmunology [377].

#### 3.4.2. Psychoneuroimmunology or Psychoimmunology

Psychoneuroimmunology (formerly known as psychoimmunology) is the study of the connections between the brain and the immune system. There are basically two major communication networks in the brain—the neurotransmitter system and the immune system. Although the blood–brain barrier is a barrier to some things, it is not an absolute barrier to the immune system. Immune activity in the body releases cytokines, chemokines, antibodies, and other substances that impact immune activity in the brain [378,379].

Infections can have several biochemical effects. One of the most significant effects relevant to mental illness is the effect of inflammation upon the kynurenine pathway. This is a pathway that converts tryptophan into serotonin and melatonin. Chronic infections that do not result in adaptive immunity can instead provoke persistent inflammation. When the brain is exposed to an inflammatory state, there is an increase in an enzyme, indoleamine 2,3-dioxygenase (IDO), that shifts the conversion of tryptophan away from serotonin, melatonin, and kynurenic acid (a neuroprotective compound) and instead pushes the conversion to quinolinic acid, which is a neurotoxin and an N-methyl-D-aspartate (NMDA) agonist [380]. Therefore, ongoing chronic inflammation switches the brain from making necessary neurotransmitters, neurohormones, and neuroprotective substances toward self-destructive activity.

Infections in the body can therefore have immune-mediated effects upon the brain through cytokine and biochemical effects. These changes can result in the dysfunction of limbic and paralimbic brain circuits, impairing emotional functioning and contributing to psychiatric symptoms and illnesses [381,382,383,384,385,386,387].

#### 3.4.3. Clinical Presentation Variability

It is clear from clinical observations of all infectious diseases that the same infection can have a very different presentation in different individuals. This is the result of a combination of different host and pathogen considerations. There are many genetic and other susceptibility and resistance contributors. Microbial variables can include the load of organisms, pathogen strain variability, and coinfections. Host variables include age, genetic, and other susceptibilities.

#### 3.4.4. Infections Early in Life with Later Consequences

Accumulating evidence supports the concept that infections early in life may play a role in the later development of mental disorders. Infections with herpes simplex virus, cytomegalovirus, rubella, and *Toxoplasma gondii* during the prenatal period are examples of infections that can result in fetal neurodevelopmental abnormalities. These can include structural abnormalities, such as hypoplasia of different areas of the brain, as well as functional problems. The resultant cerebral dysfunction can manifest as behavioral issues, learning problems, autistic spectrum disorders, or mental retardation [9,105].

One population-based cohort study utilizing a large, well-documented Australian database looked at the relationship between early exposure to infection between birth and 4 years and the subsequent development of mental disorders in children aged 5–13 years. The authors found that there was a positive correlation between the rates of childhood mental disorders and infection in the first four years of life. The authors found a moderate association of infection during this time period with autistic spectrum disorder and other developmental disorders, as well as externalizing disorders. In addition, there was a smaller but significant association with internalizing disorders [388].

A review of a nationwide Danish register-based cohort of over one million children born between 1995 and 2012 attempted to determine whether there was any association between hospitalization for infection and the later development of mental illness. The investigators found that youths who were medically hospitalized with infectious illnesses have an 84% increased risk of later developing a mental disorder. The most common mental disorders found included schizophrenia, obsessive–compulsive disorder, tics, attention-deficit hyperactivity disorder, oppositional defiant behavior, conduct disorder, personality and behavior disorders, autism, and mental retardation [389]. 

In another study, children with autism were found to have substantially greater odds of neonatal and early childhood infections when compared to children with other developmental disorders and healthy controls [136].

In summary, there is increasing evidence that the impact of infections early in life may be an important contributor to the later development of a mental illness. The various mechanisms by which this occurs require further study.

### 3.5. Clinical Considerations: Assessment

The standard of care in medicine has always been the detailed clinical evaluation. Like other illnesses, the search for the diagnosis and cause of a condition may be initiated by using a screening assessment followed by a thorough history, a review of systems, a comprehensive psychiatric clinical exam, a mental status exam, a neurological exam, and a physical exam relevant to the patient’s complaints. Laboratory or other testing may be ordered based upon the clinical assessment. The diagnosis and the cause of the condition are determined based on a knowledge of the medical literature, clinical judgment, pattern recognition, and a knowledgeable interpretation of all clinical findings [10,390].

#### 3.5.1. Clinical Assessment

The nature of the clinical assessment to determine whether microbes may be causing mental illness is an emerging field. Since infections that contribute to mental illness often cause multisystem illness, a comprehensive multisystem assessment can be helpful in determining the diagnosis and the pathophysiology. It can be challenging to differentiate between psychosomatic, somatopsychic, and multisystem illnesses and medical uncertainty [391]. Two assessments were previously developed for Lyme disease [392,393]. The General Symptom Questionnaire (GSQ-30) was developed and validated to fill the need for a brief patient-reported measure of multisystem symptom burden, and it can be useful in both clinical and research settings [394]. A multisystem assessment has been developed for Lyme borreliosis with a particular emphasis upon neuropsychiatric symptoms, which could be adapted for the assessment of any infection(s) causing mental illness. This assessment is readily available and includes screening questions, three clinical assessment forms (24-item, 61-item, full assessment), and a coinfection screen [10] (Supplementary S2 and S3).

#### 3.5.2. Laboratory and Other Diagnostic Testing

In addition to a thorough history and physical exam, a number of diagnostic tools can assist the assessment. This includes laboratory testing, structural and functional brain imaging, and neuropsychological testing. The sensitivity and specificity of laboratory testing of body fluids (blood, cerebrospinal fluid, urine) and tissue for the myriad of microbes that are associated with mental illness vary over a wide spectrum. Some are generally considered very accurate and extremely helpful in determining the presence or absence of a pathogen. On the other hand, laboratory assays are presently available only for some microbes, and their interpretative criteria have significant limitations. While serologic testing is commonly used for the lab diagnosis of microbes associated with mental illness, the detection of an antibody response to a pathogen can provide evidence of past exposure and infection but does not necessarily indicate an active, ongoing infection. At the same time, the presence of an infection does not alone prove that an infectious agent caused or contributed to any given psychiatric symptom. Psychiatric and other late-stage manifestations of an infection are more likely to occur when an infection is not adequately diagnosed and/or treated in the earlier stages of infection. A common error contributing to a lack of diagnosis or late diagnosis is confusing public health surveillance criteria with diagnostic criteria. As emphasized by the US CDC, “surveillance definitions are designed to study and identify trends in a population… Alternatively, clinical diagnoses are patient specific. Unlike surveillance definitions, ALL available diagnostic data are considered in a clinical diagnosis, including additional clinical, epidemiological and laboratory data not used for national health system surveillance. Therefore, a clinical diagnosis may be made even when a surveillance definition may not be met and vice versa is also true. Failure to meet a surveillance definition should never impede or override clinical judgment during diagnosis, management, or treatment of patents [sic]” [395]. Future testing may be more dependent upon metabolomics, the measuring and assessing of metabolites to provide specific metabolic profiling of biological fluids to help identify biomarkers for infectious disease diagnosis [263] as well as other “-omics” testing, such as proteomics, transcriptomics and metagenomic next-generation sequencing.

### 3.6. Treatment Options

Treatment must be individualized, and multidisciplinary approaches are often beneficial. Treatment can be divided into three basic areas—treatment of the infection(s) or other contributor(s), immune interventions, and treatment of the resulting symptoms and any other potential contributor(s). Initial subsequent interventions are dependent upon an understanding of the pathophysiological process, how the disease contributors interact, and the primary driver of disease perpetuation and progression.

When a patient has an inadequate response to psychotropics and infection is a possibility, antimicrobials are a consideration. When directly treating an infection, the choice of antimicrobial(s) is dependent on the microorganism, its expected or laboratory-obtained antimicrobial sensitivities, the location of the infection (e.g., CNS vs. non-CNS infection), and the medication’s adverse effect profile. It is important to note that many psychiatric conditions in which microbes may play an etiologic role are considered to be due not to a direct CNS infection but rather to a non-CNS infection that generates a pathogenic immune response with deleterious CNS effects. Antimicrobial treatment may be associated with a Jarisch–Herxheimer reaction, in which there may be a transient exacerbation of symptoms associated with the infection, including neuropsychiatric symptoms [12]. If there is a plausible mechanism for a non-infection environmental contributor, e.g., environmental toxin exposure, the mitigation of ongoing exposure, if any, to the environmental toxin and other measures to limit toxin exposure must be considered.

When immune-mediated symptoms are present from an active infection, a prior infection, or some non-infection immune provocation, consider immune-modulating interventions. With immune interventions, the choice of treatment is dependent upon whether or not there is immune suppression, excessive inflammation, autoimmunity, and/or a failure of adaptive immunity.

When symptoms such as chronic stress and sleep deprivation contribute to compromised immunity, consider symptomatic treatment. Regarding symptomatic treatment, it is necessary to perform a comprehensive clinical examination and then make a list with the patient, ranking which symptoms are the most severe and which symptoms most impede recovery. It is important to consider how symptoms interact with each other. This can help determine which symptoms are the most critical in contributing to disease perpetuation and progression. This type of reflection will help determine which symptoms to treat and in which order.

When a patient has a relapse, treatments that have been effective in the past are a consideration. When a patient is treatment-resistant, treatments that have not been used in the past are a consideration. Constant treatment revisions are needed depending upon whether the patient is improving or showing further disease progression. What has initially caused a condition may be different from what perpetuates that same condition.

From treating thousands of patients over decades, the authors (RB, RG) have found that the symptoms that usually need to be treated first are non-restorative sleep and the symptom(s) that cause the greatest chronic stress in the patient. Non-restorative sleep is often associated with fatigue and cognitive impairments, sometimes called “the terrible triad” [9,396]. A lack of restorative sleep is particularly significant in causing immune dysfunction, the failure of adaptive immunity, and disease progression [371,397,398]. In addition to non-restorative sleep, other symptoms frequently causing chronic stress in the patient include emotional symptoms (depression, anxiety, depersonalization, mood swings, psychosis, intrusive symptoms), chronic pain (headaches, neuropathy, radiculopathy, musculoskeletal pain), multisystem somatic symptoms (neurological, gastrointestinal, dysautonomia, cardiac, genitourinary, etc.), and addictive disorders [10]. 

Education to help the patient, the caregivers, and those in the support circle better understand the condition is always a critical component of treatment.

Successful psychiatric management can sometimes result in a reduction in infection. On the other hand, a successful reduction in infection can sometimes result in reduced psychiatric symptoms. Our current technological limitations prevent us from being sure that all pathogens have been eradicated. After stabilization, constant vigilance is needed to be alert to the possibility of a relapse that would require additional treatment.

### 3.7. Healthcare Delivery Issues

Adequately understanding the association between microbes and mental illness is one challenge, but using these insights to sufficiently impact the healthcare delivery system is an additional challenge. Our healthcare system is not structured to readily adapt to new approaches to emerging diseases. Super-specialization, a silo mentality, the rigidity of computerized medical electronic systems, insurance company criteria, and financial limitations impair our ability to adequately approach multisystem illness with complex infectious causes and psychiatric manifestations. The education of healthcare providers is a first step, with curricula addressing these issues in medical schools, residency programs, and allied healthcare programs. Multidisciplinary cooperation, especially between psychiatrists, psychoimmunologists, and infectious disease specialists, is needed [399].

### 3.8. Examples of Microbes Associated with Mental Illness

Five infectious diseases described as examples of microbes associated with mental illness are syphilis (a sexually transmitted spirochetal disease); toxoplasmosis (a zoonotic parasitic disease caused by *Toxoplasma gondii*); COVID-19 (a respiratory-transmitted viral disease); Lyme borreliosis and associated infections (zoonotic vector-borne disease); and group A beta hemolytic streptococcal infections and PANDAS/PANS (an autoimmune disease induced by infection and other provocations).

#### 3.8.1. Syphilis

Syphilis is caused by *Treponema pallidum* and is a historical example of a sexually transmitted infection that can cause psychiatric disease. The spirochete can survive in a host for decades with gradually increasing and expanding general medical and psychiatric symptoms. Before the introduction of penicillin, there were many patients in psychiatric institutions diagnosed with general paresis of the insane, a form of late neurosyphilis. The mental symptoms caused by syphilis include a wide variety of psychiatric syndromes, including dementia and other cognitive impairments, psychosis, and mood disorders [400]. With proper diagnosis and antibiotic therapy in its early stages, syphilis is now readily treated, thereby preventing the development of late-stage complications [401]. Noguchi and Moore’s demonstration in 1913 of spirochetes in the brains of patients with general paresis provided proof of a psychiatric disease associated with chronic meningoencephalitis from a persistent smoldering syphilis infection in brain tissue [20,402]. The course of syphilis in its various stages is shaped by the host’s immune status and immune response to spirochetal infection. Indeed, much of the pathology of this disease is felt to be due to the host inflammatory reaction to the infection rather than direct damage by spirochetes [403,404]. While late-stage neurosyphilis is, fortunately, rare, there remains a lack of consensus on its long-term outcomes with antibiotic treatment [405,406,407,408]. An important consideration with the initiation of antibiotic therapy is the potential occurrence of a Jarisch–Herxheimer reaction, which is a temporary exacerbation of underlying symptoms attributed to the antimicrobial killing of the pathogen with the release of toxic and inflammatory mediators. Herxheimer reactions historically described in neurosyphilis patients include the exacerbation of psychosis, seizure development, and the development of suicidal and violent behavior [220,409].

The history of syphilis contains a lesson in medical ethics. Penicillin was discovered in 1928. The United States Public Health Service began the Tuskegee experiment in 1932, which studied the natural course of syphilis in the study participants. Penicillin was found to be effective in treating syphilis in the early 1940s, and its use expanded during the 1940s. Penicillin treatment was withheld from the study subjects until the 1970s, with subsequent severe syphilis-related neuropsychiatric and physical complications, as well as fatalities, in many of the study participants [410]. Better Institutional Review Board oversight exists over such experiments today. However, this experiment raises one of the ethical questions that scientists struggle with: how much intervention is ethically too little or too much? At one time, penicillin was an innovative and unproven treatment for syphilis: later, it became the standard of care. There are many proposed treatments for COVID-19, Lyme disease, and other infections with physical and psychiatric consequences that may be seen as innovative by some and unproven or disproven by others. Given the risks of long-term somatic and mental sequelae from the inadequate treatment of some infections, the consideration of the risk/benefit ratio is mandatory when evaluating interventions.

#### 3.8.2. Toxoplasmosis

Toxoplasmosis is caused by Toxoplasma gondii and is a well-recognized parasitic zoonotic disease model for a parasite that can be found in the brain associated with mental illness. Some of these effects are possibly mediated by increased dopamine and decreased tryptophan. Toxoplasmosis is a disease in which a microbe completes different parts of the life cycle in different hosts. Based upon the Manipulation Hypothesis, *Toxoplasma gondii* manipulates the behavior of the host in a manner that makes the host (i.e., mouse) less fearful and more susceptible to predation by a larger mammal (i.e., the cat). This change increases the probability of transmission from an intermediate host to a definitive host [411]. Non-human mammals are the more common part of this microbe’s normal life cycle. However, humans may sometimes be accidental hosts in this zoonotic cycle. When these diseases are considered from the perspective of a zoonotic process, there may be adaptive value for the parasite to cause the host to become more aggressive, more sexually aggressive, more predatorial, and/or more susceptible to predation. As a zoonotic disease, latent *Toxoplasma gondii* infections are prevalent in humans throughout the world. Many infected individuals can have no or minimal symptoms, but the parasite can also result in psychopathology in humans [412]. The resultant psychopathology includes personality changes, mental illnesses, suicidal and homicidal behavior, schizophrenia, and auto and workplace accidents, which, collectively, can be indirectly responsible for hundreds of thousands of deaths [88,95,156,198,199,200,201,217,254,269,413,414,415].

Some studies have shown different personality changes in men vs. women. One study found that infected men were more likely to disregard rules and were more expedient, suspicious, jealous, and dogmatic, while infected women were more warm-hearted, outgoing, conscientious, persistent, and moralistic [416].

High titers of *Toxoplasma gondii* are associated with a greater propensity for suicidal behavior [417]. In a sample of 20 European nations, the prevalence of the brain parasite *Toxoplasma gondii* was positively associated with national suicide rates for men and women [418]. *T. gondii* seropositivity is also associated with a seven-fold greater risk of self-directed violence [419,420]. In a sample of 20 European nations, the prevalence of *Toxoplasma gondii* was positively associated with higher national homicide rates, which further amplified the research indicating a positive association of *Toxoplasma gondii* with suicide rates [421].

#### 3.8.3. COVID-19 (Coronavirus Disease 2019)

COVID-19, caused by severe acute respiratory syndrome coronavirus 2 (SARS-CoV-2), is a viral model of a microbe that, in some persons, plays an important role in the subsequent development of a mental illness. Since the identification of SARS-CoV-2 as the cause of an outbreak of pneumonia in Wuhan, China, in December 2019 and its subsequent rapid spread to other countries worldwide, information in this area has been rapidly evolving. In some, COVID-19 is a mild, at times subclinical infection, while in others, it can be severe or even fatal. Neuropsychiatric manifestations of acute infection include delirium, confusion, emotional disturbances, and psychosis [422]. A subset of patients experience residual sequelae after acute infection, including manifestations particularly relevant to psychiatry. Known as post-acute sequelae of COVID-19 (PASC) and also referred to as “long COVID”, these symptoms can resolve with the passage of time in some individuals while remaining chronic in others. In patients with chronic symptoms, there are similarities to other post-acute viral syndromes, such as Chronic Fatigue Syndrome/Myalgic Encephalomyelitis [423,424]. Symptoms can occur in multiple organ systems. Persistent symptoms may include fatigue, post-exertional malaise, and multiple emotional and cognitive impairments (memory impairment, attention deficits, cognitive difficulties, executive dysfunction) [422]. In addition to cognitive deficits, mental symptoms include anxiety, depression, mood swings, bipolar/manic episodes, obsessive–compulsive disorders, posttraumatic stress, new-onset psychosis, sleep disturbances, substance use disorders, suicidality, and symptom constellations consistent with pediatric acute-onset neuropsychiatric syndrome (PANS; see Section 3.8.5 for additional PANS information) [5,50,81,82,83,84,85,422,425,426,427,428,429,430,431,432]. One retrospective cohort study using electronic health record data comprising 81 million patients in healthcare organizations primarily in the US showed that the risk of common neuropsychiatric disorders (mood disorders, anxiety disorders) returned to baseline in 1–2 months, but there remained an elevated risk of psychotic disorders, cognitive deficit, dementia, and seizures at two years of follow-up [83]. In contrast, another study using data from the large US Veterans Health Administration national healthcare database showed, at one year of follow-up, an increased risk of an array of incident mental health disorders, including anxiety, depression, sleep disorders, stress and adjustment disorders, opioid and substance use disorders, and neurocognitive decline [426].

The proposed mechanisms for PASC [433,434,435,436,437,438] include persistent reservoirs of pathogens or pathogen remnants (e.g., SARS-CoV-2 structural proteins such as spike protein or spike protein fragments such as the S1 subunit); the development of autoimmunity due to molecular mimicry between pathogen and host proteins; the reactivation of other latent pathogens; dysbiosis from SARS-CoV-2–host-microbiome interactions; and resultant organ system or tissue damage from the infection and associated immunopathology, including vasculopathy, coagulopathy, and clotting issues. These potential contributors may act independently or in combination to cause persistent symptoms. 

#### 3.8.4. Lyme Borreliosis and Associated Diseases

Lyme borreliosis is a prime example of a vector-borne (tick) zoonotic infection that can have neuropsychiatric manifestations. An estimated 476,000 Americans yearly are diagnosed and treated for Lyme disease, making it the most common vector-borne disease in the US [439]. What is now commonly called Lyme disease was first described in Europe before World War I. In 1970, Dr. Rudolph Scrimenti in Wisconsin reported the first case in the United States, a patient with an erythema migrans rash at the site of a tick bite, accompanied by a low-grade fever, headache, and malaise, with symptoms responding to treatment with intramuscular penicillin [440]. The disease became more widely recognized following an epidemic of arthritis in children and adults in Lyme, Connecticut, which was described in multiple published reports by Dr. Allen Steere and colleagues [441,442,443]. Dr. Willy Burgdorfer discovered the causative organism, the bacterium *Borrelia burgdorferi* [444]. Like syphilis, Borrelia burgdorferi is a complex spirochetal illness. In contrast to syphilis, it has a much more complex genome that may give it greater adaptive capability to survive under different conditions in different hosts, namely, cold-blooded ticks versus warm-blooded vertebrates [445].

The nomenclature regarding *Borrelia burgdorferi* infection and Lyme disease is confusing and needs clarification. Lyme disease or Lyme borreliosis is the disease resulting from *Borrelia burgdorferi* sensu lato (Bbsl) infection. Included within Bbsl are three major *Borrelia* species that cause Lyme disease: *Borrelia burgdorferi* (also called *Borrelia burgdorferi* sensu stricto) causes the disease in the US, while *Borrelia afzelii* and *Borrelia garinii* are the predominant species causing Lyme borreliosis in Europe and Asia.

Similar to syphilis, Lyme disease is a complex illness with multi-symptom, multisystemic manifestations that are commonly described as presenting in stages. Early localized disease symptoms include a skin lesion (erythema migrans, or EM) that may or may not be accompanied by constitutional symptoms. Early disseminated disease includes multiple EM lesions and neurologic and cardiac manifestations. Late disseminated disease includes joint and neurologic manifestations. Some less common organ system manifestations, such as ocular involvement, can represent early or late disease. The psychiatric symptoms of Lyme disease are mostly late-stage symptoms [10].

Since many healthcare professionals are unaware of the full range of potential manifestations of Lyme disease, as well as the limited sensitivity of commonly used laboratory tests and interpretation criteria, many early cases are undiagnosed and untreated. These frequently avoidable errors can result in progression to late-stage disease with significant neuropsychiatric symptomatology [9,10].

Lyme borreliosis/tick-borne disease is associated with almost any psychiatric syndrome listed in the American Psychiatric Association *DSM5-TR* [9,164,312] (Supplementary S1). The neuropsychiatric symptoms seen with Lyme/tick-borne disease include developmental disorders, autism spectrum disorders, schizoaffective disorders, bipolar disorder, depression, anxiety disorders (panic disorder, social anxiety disorder, generalized anxiety disorder), posttraumatic stress disorder, eating disorders, sleep disorders, addiction, suicide, violence, anhedonia, depersonalization, dissociative episodes, derealization, hallucinations, intrusive symptoms, and vegetative functioning impairments. Possible cognitive impairments that can be precipitated include dementia and impairments in attention span, memory, processing, and executive functioning [9,10]. 

A large seroepidemiology survey in the Czech Republic reported *Borrelia burgdorferi* seropositivity in approximately 1/3 of psychiatric patients, a rate that was 1.7-fold higher than in matched healthy controls [446]. In the peer-reviewed medical literature, there are currently 501 references demonstrating an association between Lyme borreliosis/tick-borne disease and neuropsychiatric diseases and 88 demonstrating an association with dementia (in Supplementary S1, Peer-Reviewed Evidence of Lyme Borreliosis/Tick-Borne Disease Associated with Psychiatric Symptoms).

There are two broad postulated or demonstrated mechanisms by which late Lyme neuroborreliosis might lead to neuropsychiatric symptoms. First, there can be direct injury from CNS infection, i.e., Lyme encephalitis or meningoencephalitis. This is an infection within the parenchyma of the brain, associated in its infiltrative form with strong perivascular lymphoplasmacytic infiltrates, vasculitis, and microgliosis and in its atrophic form with cortical atrophy, gliosis, and dementia [402]. Second, infections outside the central nervous system (CNS), or perhaps a “smoldering” CNS infection, can have indirect immune and toxic effects upon the CNS through a variety of mechanisms, including molecular mimicry leading to autoantibody formation and cellular immune responses against host neuronal structures; altered metabolism of tryptophan resulting in the decreased production of serotonin and the increased production of neurotoxic and neuroexcitatory kynurenine metabolites; and consequent oxidative stress, neuronal excitotoxicity, changes in homocysteine metabolism, and mitochondrial dysfunction [12,357,358,363,365,366,372,374,447].

In 75% of chronic Lyme disease patients with neurocognitive and/or mood dysfunction that impaired their daily living activities, single-photon emission computed tomography brain imaging demonstrated perfusion deficits to various areas of the brain, most notably the frontal, temporal, and parietal lobes, Patients considered to be seropositive and those considered seronegative had similar rates and severity of perfusion defects. Antibiotic treatment, especially agents with intracellular-penetrating activity, resulted in the resolution or improvement of abnormalities in 70% of patients over a 1- to 2-year period [448].

Manifestations of Lyme disease that continue or recur for an extended period (more than 6 months) after commonly used antibiotic therapy are frequently referred to as either chronic Lyme disease [449,450] or post-treatment Lyme disease syndrome [451]. In their common historical use, these terms tend to connote different viewpoints on the likely etiology of persistent symptoms. “Chronic Lyme disease” commonly connotes that persistent symptoms could be due to an ongoing, active *Borrelia* infection [450]. As originally defined, “post-treatment Lyme disease syndrome” consists of persistent or recurrent symptoms of pain, fatigue, or cognitive issues lasting more than 6 months after treatment with a 2–4-week course of antibiotics and is considered to be of unknown etiology, although one postulated view is that the symptoms could be autoimmune in nature [338,451]. Importantly, these two explanations for persistent symptoms (ongoing, active infection vs. autoimmunity) need not be mutually exclusive. The latest US Tick-Borne Disease Working Group 2022 Report to Congress uses the term “persistent Lyme disease/chronic Lyme disease” [452] while neutrally recognizing the existence of divergent views on the cause of persistent symptoms.

None of the three aforementioned terms address the potential presence of tick-borne or other coinfections impacting disease presentation. Many other pathogens may be introduced in the tick bite in addition to *Borrelia*. Evidence indicating the presence of different *Bartonella* spp. DNA in various types of tick species from diverse geographic locations has also been reported [453]. Common coinfections besides *Borrelia* that are particularly relevant to neuropsychiatric symptoms include different species of *Babesia* [91,105,106,207] and *Bartonella* [25,26,28,105,106,162,163,207,208,221,222,223,224]. 

While the vector competence of *Ixodes scapularis* ticks for *Bartonella* transmission to a human host has yet to be definitively demonstrated and requires further study, *Bartonella* transmission by *Ixodes ricinus*, the tick vector for Lyme disease in Europe, has been documented in mice [454]. Furthermore, multiple other insect vectors are known or suspected to transmit *Bartonella* to humans [453]. Overall, the recognition of coinfections and their potential role in complex cases of Lyme disease is an emerging field of research. Presently, there are many recognized and yet-to-be-recognized tick-borne microbes that may act as human pathogens [455,456,457,458,459].

Considerable confusion, controversy, and complexity surrounding Lyme disease testing contribute to many missed or late diagnoses of Lyme disease that then can manifest with significant late-stage neuropsychiatric symptomatology [9,10,460], and hence, some clarification is in order. Most Lyme disease testing today is indirect testing that detects IgM and/or IgG antibodies produced in response to *Borrelia* infection, rather than direct testing for the organism itself. The CDC recommends two-tier serologic testing, typically consisting of a first-tier enzyme immunoassay (EIA) that, if positive or indeterminate, is followed by a more specific, second-tier Western blot or immunoblot assay [461]. More recently, the CDC updated their Lyme testing recommendations to allow the use of an EIA other than a Western immunoblot assay as an alternative second-tier test [462]. However, for complex cases, as commonly occurs in patients with neuropsychiatric manifestations, Western immunoblot testing may provide valuable information by demonstrating separate highly *Borrelia*-specific antibodies, as well as the degree of expansion of the Lyme antibody response, which may provide better support for a clinical diagnosis and help delineate duration of illness [463]. The commonly used interpretation criteria for a positive IgM or IgG immunoblot, which have been widely used since the mid-1990s [461], have been critiqued as being overly restrictive [449,460,464,465]. Notably, the IgG immunoblot criteria for the diagnosis of disseminated/late Lyme disease are based on a single study that reported an overall sensitivity of 83% for their proposed criteria, with substantially lower sensitivity for the subset of patients with neurologic disease compared to those with arthritis (72% vs. 96%, respectively; these figures are calculable from study data presented in their Table 4) [466]. Similarly low or even lower sensitivity of commonly used two-tiered testing—in one study, 43% [467] and, in another study, ranging from 44 to 74% [468]—was reported by two independent research groups, both using a CDC reference panel of serum samples and the CDC-recommended test interpretation criteria. Several subsequent and oft-cited studies reported high sensitivity (97–100%) of commonly used two-tier testing and interpretation criteria in late Lyme disease [469,470,471,472], but the problematic selection bias inherent in their study design deserves commentary. Specifically, the inclusion criteria in these studies required late-disease patients to have lab confirmation, either as defined in CDC surveillance criteria at the time or even as shown by positive two-tier serology. Additionally, in the studies that specified separate numbers for arthritis vs. neurologic disease cases, there were few cases of the latter—in one study, just 11 patients with late neurologic disease [469], and in another study, only 2 patients [470]. Furthermore, in each of these two studies, half or more of the few patients with neurologic disease also had current or previously treated arthritis. Attention to such details is warranted since, as previously noted, the sensitivity of the commonly used IgG immunoblot interpretation criteria for disseminated/late Lyme disease was substantially higher in patients with arthritis compared to neurologic disease in the single study that forms the basis for those criteria [466].

A final point that deserves emphasis is that lab testing cannot be relied on alone to diagnose or exclude Lyme disease and, instead, must always be considered in the context of the whole clinical picture. Clinicians must be familiar with the full array of clinical manifestations of Lyme borreliosis, as, ultimately, Lyme disease must be diagnosed clinically on the basis of clinical history and exam findings, with lab testing regarded as supportive or not of the clinical diagnosis [473,474]. The possibility of false-negative serologic tests in early disease is well recognized, as it takes time for the immune system to respond to infection with Borrelia-specific antibody production. In late Lyme disease, false-negative serologies can also occur due to a variety of reasons [475,476]. Indeed, seronegativity is well documented in patients with late Lyme borreliosis, including PCR- or culture-confirmed disease [477,478,479,480]. Conversely, false positives can be seen due to cross-reactivity in patients with other spirochetal diseases or clinical conditions, such as autoimmune diseases [475]. Additionally, in some Lyme-endemic areas in the US or Europe, background seropositivity rates as high as 11–20% have been reported in healthy blood donors or general outpatients not being seen for tick-borne disease testing [481,482,483,484,485]. In summary, laboratory testing alone cannot be the sole basis for determining whether any infection did or did not play a role in the development of any particular psychiatric symptom or syndrome. Instead, there must be reliance on the total clinical assessment [7,8,9,10].

#### 3.8.5. Group A Streptococcal Infections, PANDAS, and PANS

An area of active investigation for more than two decades that helps demonstrate a connection between microbes and mental illness is the field of pediatric autoimmune neuropsychiatric disorders associated with streptococcal infections (PANDAS). The illness follows a group A streptococcal (GAS) infection, such as pharyngitis, scarlet fever, or an anal streptococcal infection. Subsequently, the individual experiences a sudden onset of obsessive–compulsive symptoms, a tic disorder, or both [486]. Accompanying the psychiatric symptoms are new-onset neurologic abnormalities, such as physical hyperactivity or unusual, jerky involuntary movements. In addition, there are a variety of associated symptoms, including mood changes, increased irritability, sleep difficulties, separation anxiety symptoms, increased day or nighttime urination, joint pains, and motor skill changes, such as a decrement in handwriting [487].

The proposed etiological mechanisms for PANDAS include molecular mimicry and the development of autoimmunity. These processes play an important role in the development of cardiac valvular disease and Sydenham’s chorea, which can occur in individuals suffering from post-Streptococcal Rheumatic Fever [488]. In PANDAS, the proposed mechanism of causation involves antibodies that react to components of the strep bacteria and subsequently cross-react with similar molecules located in the child’s basal ganglia [489]. Experiments using mouse models have found that group A streptococcus infection induces a strong Th17 immune response in the nasal-associated lymphoid tissue (NALT), the murine equivalent of human palatine tonsils [490]. GAS-specific Th17 cells have also been shown to exist in the tonsils of people naturally exposed to group A strep [490]. In a mouse model, multiple GAS challenges promote the migration and persistence of GAS-specific Th17 cells to the brain, leading to blood–brain barrier breakdown and autoantibody access to the CNS [490].

Th17 memory cells help control extracellular bacterial infections and fungi at mucosal surfaces by recruiting and activating myeloid cells (neutrophils) [491] but also play a role in the generation of the immunopathology characteristic of autoimmune conditions such as rheumatoid arthritis and multiple sclerosis [492,493]. In a murine model of PANDAS, evidence supports the action of Th17 cells releasing the cytokine IL-17A, which is thought to play an important role in the entry of immune system cells into the CNS [494]. There, it leads to the disruption of healthy brain cells, eventually resulting in the development of neuropsychiatric symptoms. One hypothesis is that the olfactory bulb’s proximity to the nasal mucosa makes it a susceptible target for insult and infection of the brain [495]. Studies in the literature support the use of the olfactory route by many viruses and bacteria to create an infection within the brain [496]. However, in the case of PANDAS, there is no evidence of streptococcal entry across the blood–brain barrier and into the brain [490]. Recent work reported in preprint form by Agalliu and colleagues demonstrates that two Th17 effector cytokines, IL-17A and GM-CSF, differentially promote blood–brain barrier dysfunction and the microbial expression of interferon-response and chemokine genes in a murine model of intranasal GAS infections [497]. The brain microglia release a variety of chemokines that might act as potential biomarkers of the illness. Collectively, the evidence supports the idea that PANDAS is a form of infection-induced immune-mediated encephalitis.

Since the original description of PANDAS, it has subsequently become recognized as part of a broader category called pediatric acute-onset neuropsychiatric syndrome (PANS) [243,498]. Unlike PANDAS, the name PANS recognizes that the neuropsychiatric and other manifestations of this syndrome can be triggered by not just streptococcal infections but also a variety of other infections [243,499] (e.g., *Mycoplasma* [263], *Borrelia* (Lyme disease) [500], *Bartonella* [162], SARS-CoV-2 [82,425,437]), as well as, potentially, non-infectious triggers, such as environmental exposure to toxins or other inducers of inflammatory reactions [498,501]. As originally defined, the primary symptoms of PANS are the sudden onset of obsessive–compulsive symptoms or restricted eating behaviors. Additional symptoms of PANDAS and PANS include a variety of neuropsychiatric as well as somatic symptoms, including depression, anxiety, emotional lability, irritability, aggression, behavioral regression, ADHD-like symptoms, cognitive changes, sleep disturbances, and urinary frequency or enuresis [243,498,502].

## 4. Discussion

The microbial impact upon human mental functioning is more extensive than generally appreciated. Historically, there have been, and continue to be, many models to attempt to explain the causes of and contributors to mental illnesses. The increasing recognition that microbes may contribute to mental illness is expanding as clinical observations and newer technologies provide greater evidence.

A considerable amount of the literature recognizes that psychiatric conditions may be associated with infectious contributors. This is demonstrated in Table 1 and Table 2. There are multiple pathophysiological mechanisms explaining this association. The review of the five infection-associated diseases further supports the association between microbes and mental illness.

As a result, clinicians need to consider the possibility of infectious diseases in formulating a pathophysiological explanation and a differential diagnosis. Considering the complexity and interactive nature of the pathogen and host in illness, treatments including anti-infective and/or immune interventions become potential treatment options. This consideration may be of particular significance with treatment-resistant psychiatric illness.

It is important to look at the five examples of microbes associated with mental illnesses discussed in this article from a historical perspective. The effective treatment of neurosyphilis was not fully implemented for many years after the discovery of penicillin. The other four infection-associated diseases are surrounded by controversy. Will current technology facilitate more effective progress toward understanding and treating these illnesses? 

There are many obstacles preventing forward progress in recognizing the association between microbes and mental illness. These include difficulties appreciating the potential role of infections in chronic illness, failure to recognize complex disease models, the difficulty developing disease models when there are multiple variables with susceptibility and infection, pathophysiology and manifestations, failure to appreciate the role of chronic relapsing infections and complex interactive infections with psychiatric illness, a silo mentality, paradigm blindness, investments in outdated belief systems, current clinical assessment limitations, laboratory limitations, the limitations of antimicrobial and other treatments, educational limitations, and healthcare system limitations.

Potential solutions include the following: (1) expanded research into the association between microbes and mental illness; (2) a reexamination of existing paradigms to identify and correct gaps and inconsistencies; (3) improved multidisciplinary collaboration; and (4) expanded clinician and public education.

### Future Directions

Investigation and collaboration among multiple specialists are needed to further clarify and expand all of the topics that have been reviewed in this article. Table 1 can be expanded, and Table 2 can be expanded to include additional diagnostic categories. Additional research and education addressing the association between microbes and mental illness are needed. In addition, the education of healthcare providers, with curricula addressing microbes and mental illness in medical schools, residency programs, and allied healthcare programs, and multidisciplinary cooperation, especially between psychiatrists, psychoimmunologists, and infectious disease specialists, will help advance progress toward a better understanding of the etiology of mental illnesses.

## 5. Conclusions

The findings of this review support the concept that infectious diseases may have an important role in psychiatric diagnosis and treatment. Indirect mechanisms of infection, such as inflammation, neuroinflammation, autoimmunity, and other neurophysiological changes, have been implicated in the development and/or progression of some mental illnesses. The persistence of these processes may result in chronic effects on brain structure and function. A combination of these indirect actions, with direct pathophysiologic effects from infectious agents, has the potential to result in a broad spectrum of mental symptoms and illnesses. Further investigation into the association between infectious/immune processes and mental disorders may lead to greater use of antimicrobial and immune-modulating agents in the treatment of psychiatric conditions. This may prevent and reduce mental illness morbidity, disability, and mortality.

## Figures and Tables

**Table 1:** Infectious agents with potential psychiatric manifestations.

**Spirochetes**
*Borrelia burgdorferi* sensu lato (new genus name *Borreliella*) [9,10,11,12,13,15,16,17] (Supplementary S1)
*Borrelia burgdorferi* sensu stricto (Lyme disease in USA, Europe)
*Borrelia afzelii* (Lyme disease mostly in Europe, Asia)
*Borrelia garinii* (Lyme disease mostly in Europe, Asia)
Relapsing fever group (also known as relapsing fever group *Borrelia*) [18]
*Leptospira* species (leptospirosis) [19]
*Treponema pallidum* (syphilis) [20,21,22,23]
**Other bacteria**
*Actinomyces* [24]
*Bartonella henselae* and other species (cat scratch disease, bartonellosis) [25,26,27,28]
*Brucella* species (brucellosis) [29]
*Chlamydia* species [30,31]
*Coxiella burnetii* (Q Fever and “Post-Q Fever Fatigue Syndrome”) [32]
*Ehrlichia chaffeensis* (human monocytic ehrlichiosis) [33,34]
*Helicobacter pylori* [35]
*Mycoplasma pneumoniae* and other species [36,37,38]
*Rickettsia* species (spotted fever, scrub typhus, African tick bite fever) [39,40,41,42,43]
*Streptococcus pyogenes* (group A beta hemolytic strep, PANDAS, Sydenham’s Chorea, St Vitus Dance) [44]
*Tropheryma whipplei* (Whipple’s disease) [45,46]
**Viruses**
Borna disease virus [47]
Chikungunya virus [48]
Coronaviruses (other than SARS-CoV-2) [49,50,51]
Enterovirus [52,53,54]
Cytomegalovirus [55,56,57]
Epstein–Barr virus [58,59]
Tick-borne encephalitis virus [60]
Hepatitis C virus [61,62,63]
Human endogenous retroviruses [64,65,66,67] H
Human immunodeficiency virus [68]
Human T-cell lymphotropic virus type 1 [69]
Influenza virus [70]
Measles virus [71,72,73,74,75,76]
Parvovirus B19 [77,78]
Poliovirus [79]
Rubella [80]
SARS-CoV-2 coronavirus [50,81,82,83,84,85]
West Nile virus [86,87]
**Parasites** [88]
*Plasmodium* species (malaria) [89,90]
*Babesia species* (*B. microti*, *B. duncani*, *other Babesia species* (Babesiosis)) [91]
*Filaria* (filariasis) [88,92,93]
*Leishmania* species (leishmaniasis) [94]
*Toxoplasma gondii* (toxoplasmosis) [95]
*Taenia solium* (neurocysticercosis or cysticercosis) [96,97,98]
*Trypanosoma* sp. (trypanosomiasis) [88,99,100]
**Fungi**
*Aspergillus* species [24]
*Candida* [101,102]
*Cryptocococcus neoformans* (cryptococcosis) [103,104]

**Table 2:** Examples of mental conditions potentially associated with infections.

Mental Conditions	Infections	Citations
Autism spectrum disorders	*Babesia*	[105,106]
	*Bartonella*	[105,106]
	Borna disease virus (animal models)	[107,108,109,110]
	*Borrelia burgdorferi* and other tick-borne diseases	[105,106,111,112,113,114,115,116,117]
	*Chlamydia pneumoniae*	[117,118,119]
	Cytomegalovirus	[120,121,122,123,124]
	Enterovirus	[53,54]
	Fungi (Aspergillus, Candida)	[125,126,127,128,129]
	Herpes simplex virus	[130,131,132,133,134]
	Human herpes virus-6	[118,135]
	Infections in early childhood	[136,137]
	Intestinal microbiome composition changes	[138]
	Maternal infections or immune activation during pregnancy	[139,140,141,142,143,144,145]
	*Mycoplasma:* (*M. fermentans*, *M. genitalium*, *M. hominis*, *M. pneumoniae*)	[36,117,118]
	Measles virus (subacute sclerosing panencephalitis)	[135,146]
	*Plasmodium* (malaria)	[147,148,149,150]
	*Rubella* (congenital)	[80,151,152,153,154,155]
	*Toxoplasma gondii* (Toxoplasmosis)	[156]
	Varicella zoster virus	[157]
	Viral infections	[158,159,160]
Schizophrenia	*Aspergillus*	[24]
	Bacterial infections	[161]
	*Bartonella*	[25,162,163]
	*Borrelia burgdorferi* (Lyme disease)	[17,164,165,166,167,168,169,170,171]
	Borna disease virus	[172]
	*Candida albicans*	[101,102]
	*Chlamydia*, (*C. psittaci*, *C. pneumoniae*)	[31,172,173,174,175]
	Coronaviruses	[49]
	*Cryptocococcus neoformans* (cryptococcosis)	[103]
	Cytomegalovirus	[176]
	Epstein–Barr virus (EBV)	[58,177,178,179]
	Herpes simplex virus	[172,180,181]
	Human endogenous retroviruses	[64,65,172,182,183,184]
	Infections in early childhood	[137]
	Influenza virus	[185,186,187,188,189,190,191,192]
	Maternal infections or immune activation during pregnancy	[161,182,183,185,186,187,188,189,190,191,192,193,194,195]
	Measles virus (subacute sclerosing panencephalitis)	[71,72,73,74,75,76]
	Parvovirus	[78]
	Poliovirus	[79]
	Rubella	[194,196]
	*Taenia solium* (neurocysticercosis or cysticercosis)	[88,97]
	*Toxoplasma gondii*	[175,176,193,197,198,199,200,201]
	*Treponema pallidum* (syphilis)	[23,202,203,204,205]
Bipolar disorders	*Bartonella*	[206,207,208]
	*Borrelia burgdorferi*	[10,165,207,209,210,211]
	Cytomegalovirus	[57,176]
	Human endogenous retroviruses	[212]
	*Mycoplasma*	[207,213]
	Parvovirus B19	[78]
	SARS-CoV-2	[214,215]
	Tick-borne diseases	[216]
	*Toxoplasma gondii*	[176,217]
	*Treponema pallidum* (syphilis)	[205,218,219,220]
Depressive disorders	*Babesia*	[91]
	*Bartonella*	[27,28,162,208,221,222,223,224]
	*Borrelia burgdorferi*	[10,17,164,225,226,227,228,229,230,231]
	Borna disease virus	[47]
	Cytomegalovirus	[57]
	Enterovirus	[52]
	Hepatitis C virus	[61,62,63]
	Human immunodeficiency virus (HIV)	[68,232]
	Human T-cell lymphotropic virus type 1 (HTLV-1)	[69]
	Infections early in childhood	[137]
	Measles virus (subacute sclerosing panencephalitis)	[75]
	*Plasmodium* (malaria)	[233,234]
	SARS-CoV-2 and other coronaviruses:	[50,51,81,235]
	*Taenia solium* (neurocysticercosis or cysticercosis)	[96,97,98]
	*Treponema pallidum* (syphilis)	[23,205,218,220]
	West Nile virus	[86,87,236]
Anxiety disorders	*Bartonella*	[27,206,207,221,224]
	*Borrelia burgdorferi*	[10,164,211,229,237,238]
	Epstein–Barr virus	[239]
	Human T-cell lymphotropic virus type 1	[69]
	*Mycoplasma pneumoniae*	[38,207,240,241]
	SARS-CoV-2	[50,81,84,235,242]
	*Streptococcus pyogenes* (group A strep)	[240,243]
	*Treponema pallidum* (syphilis)	[205]
Suicidality	All infections requiring hospitalization (including infections requiring hospitalization for COPD)	[244]
	*Bartonella*	[162,206,208]
	*Borrelia burgdorferi*	[10,11,165,209,228,231,245,246]
	Cytomegalovirus	[57]
	Hepatitis C virus	[247]
	Herpes simplex virus type 1 (HSV-1)	[248]
	Human immunodeficiency virus (HIV)	[249]
	Influenza virus	[250]
	SARS-CoV-2	[251,252]
	*Streptococcus pyogenes* (group A Strep)	[253]
	*Toxoplasma gondii*	[254]
Aggressive or violent behavior	*Babesia*	[91]
	*Bartonella*	[25,26,162,207,221]
	*Borrelia burgdorferi*	[10,16,209,211,246,255,256]
	Encephalitis lethargica agent	[257,258]
	Hepatitis E virus	[259]
	Herpes simplex virus	[260,261]
	Infection during childhood	[262]
	Measles virus (subacute sclerosing panencephalitis)	[75]
	*Mycoplasma*	[38,263]
	Parvovirus	[77]
	*Plasmodium* (Malaria)	[264,265]
	Rabies virus	[266,267,268]
	SARS-CoV-2	[81]
	*Streptococcus pyogenes* (group A Strep)	[208,241]
	*Toxoplasma gondii* (toxoplasmosis)	[269]
	*Treponema pallidum* (syphilis)	[204,270,271]
	Viral encephalitis	[272]
	Animal models of infections associated with aggression include *Borrelia burgdorferi* in dogs, *Bartonella henselae* in dogs, *B. henselae* in horses, *B. burgdorferi* postulated in chimpanzee in lay news, rabies virus in multiple animal species, and gut microbiota in dogs, horses, and pigs.	[273,274,275,276,277,278,279,280,281,282,283]

## Data Availability

Data are contained within the article and Supplementary Materials.

## Supplementary Materials

The following supporting information can be downloaded at https://www.mdpi.com/article/10.3390/healthcare12010083/s1: Supplementary S1: Peer-Reviewed Evidence of Lyme/Tick-Borne Disease Associated with Psychiatric Symptoms; Supplementary S2: Lyme Disease Screening Assessment; Supplementary S3: Coinfection Screen.
